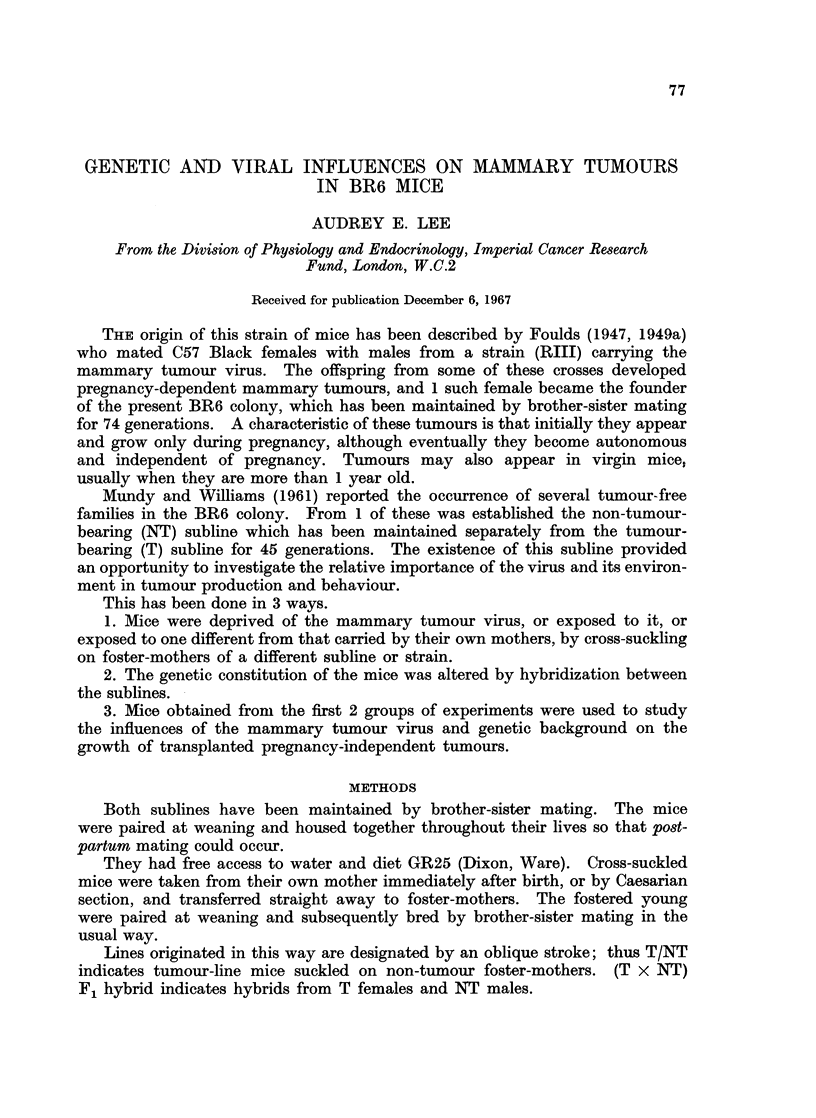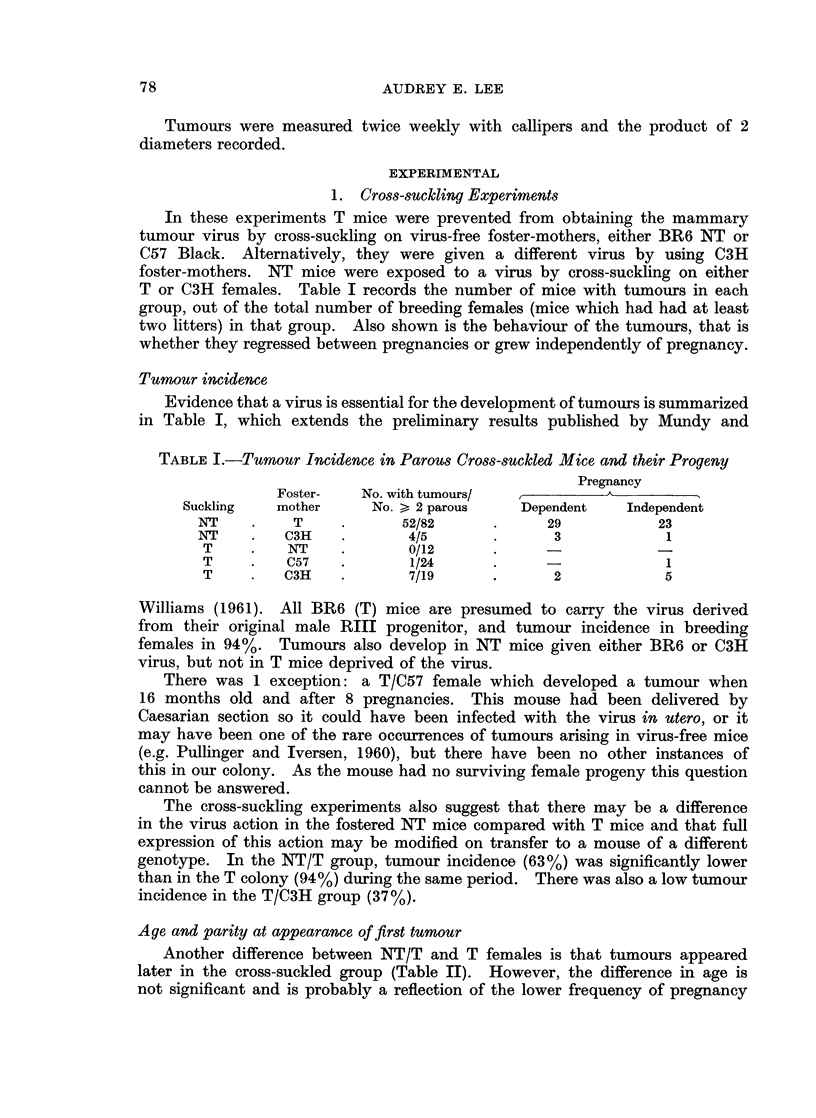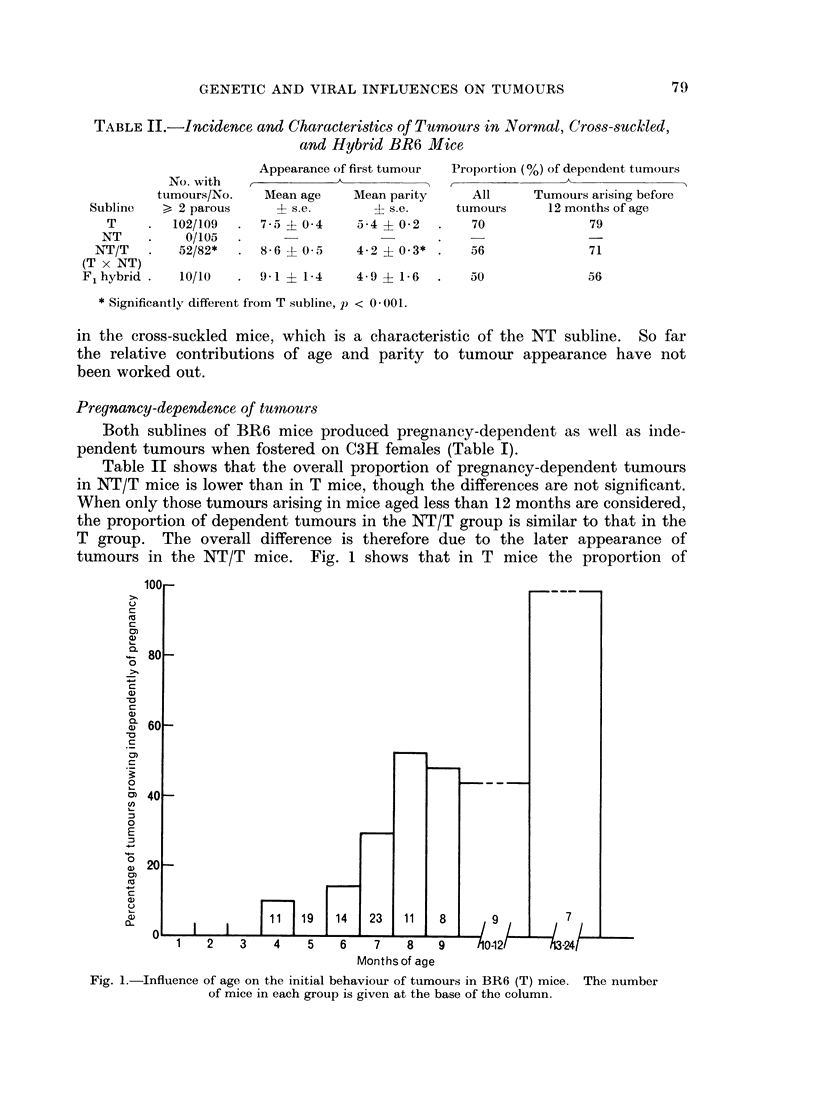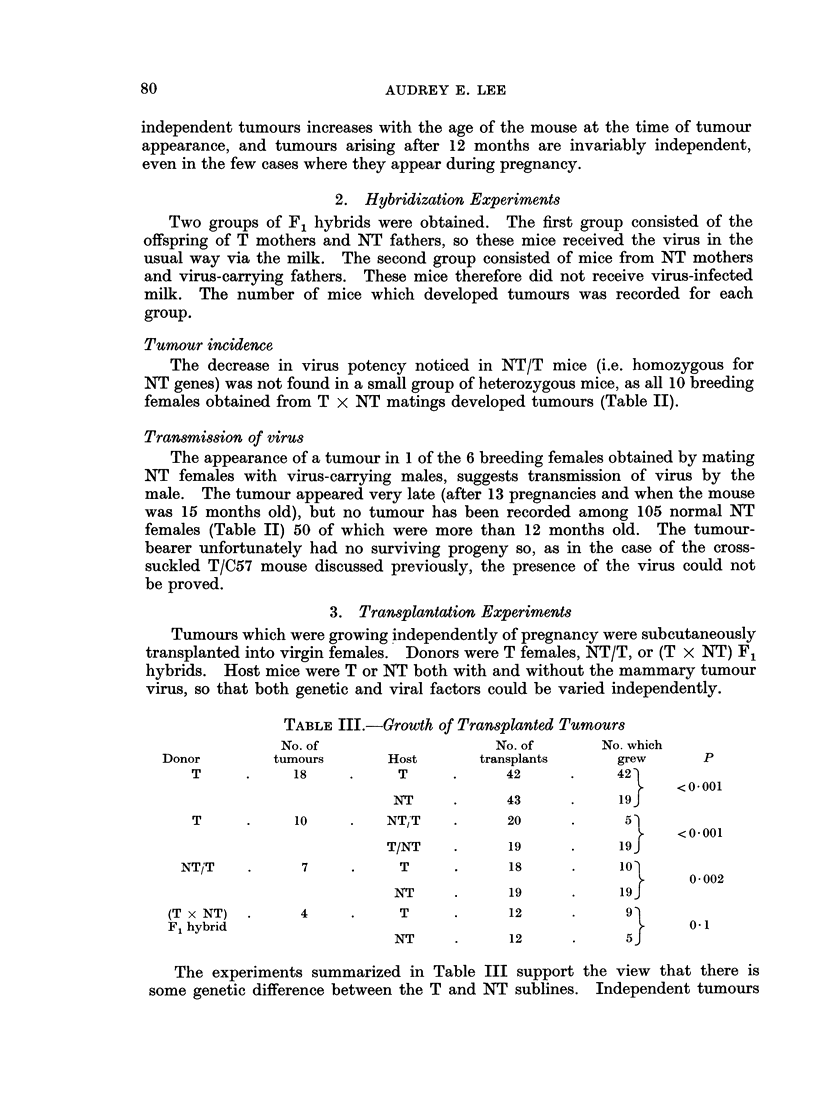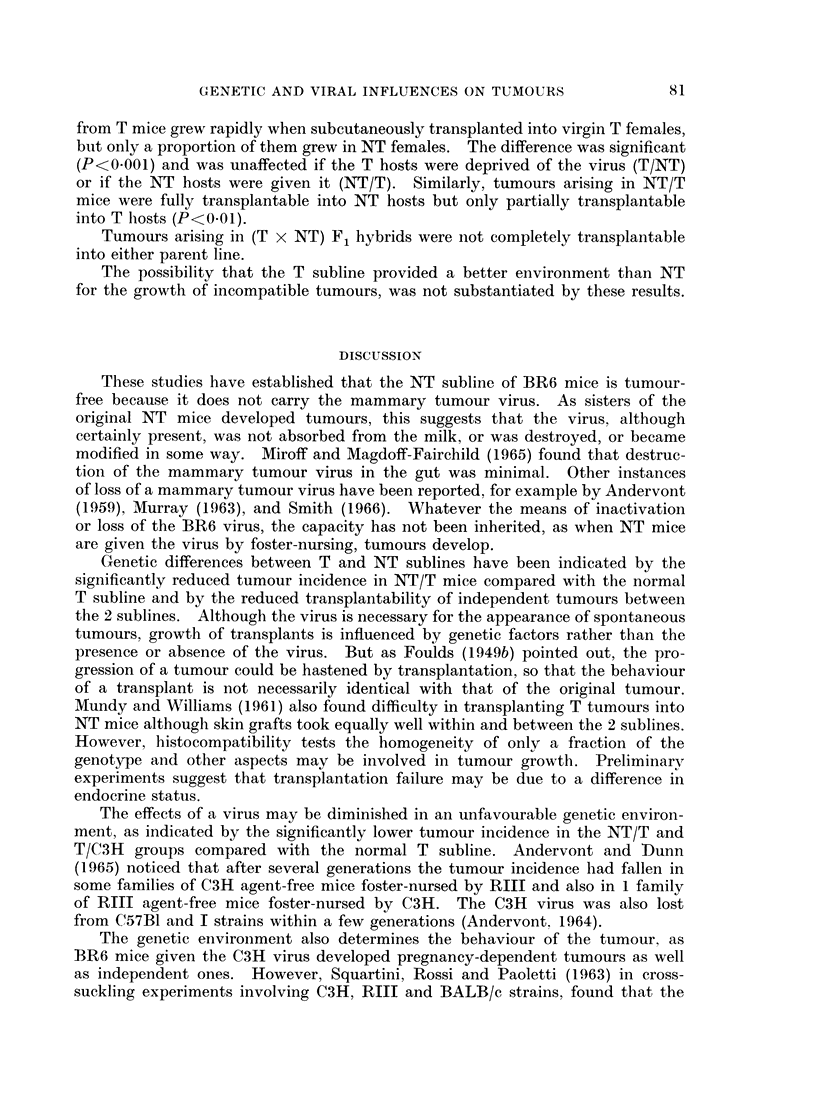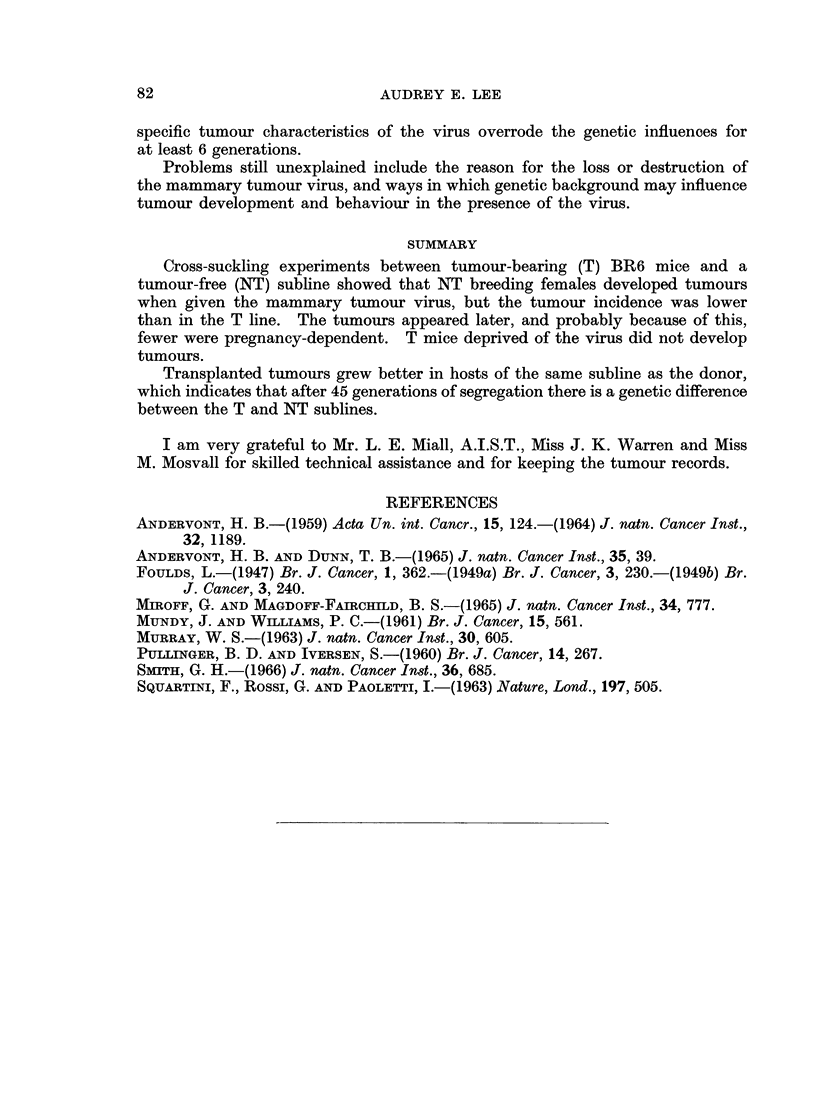# Genetic and viral influences of mammary tumours in BR6 mice.

**DOI:** 10.1038/bjc.1968.11

**Published:** 1968-03

**Authors:** A. E. Lee


### GENETIC AND VIRAL INFLUENCES ON MAMMARY TUMOURS IN BR6 MICE

AUDREY E. LEE

*From the Division of Physiology and Endocrinology, Imperial Cancer Research Fund, London, W.C.2*

Received for publication December 6, 1967

THE origin of this strain of mice has been described by Foulds (1947, 1949a) who mated C57 Black females with males from a strain (RIII) carrying the mammary tumour virus. The offspring from some of these crosses developed pregnancy-dependent mammary tumours, and 1 such female became the founder of the present BR6 colony, which has been maintained by brother-sister mating for 74 generations. A characteristic of these tumours is that initially they appear and grow only during pregnancy, although eventually they become autonomous and independent of pregnancy. Tumours may also appear in virgin mice, usually when they are more than 1 year old.

Mundy and Williams (1961) reported the occurrence of several tumour-free families in the BR6 colony. From 1 of these was established the non-tumour-bearing (NT) subline which has been maintained separately from the tumour-bearing (T) subline for 45 generations. The existence of this subline provided an opportunity to investigate the relative importance of the virus and its environment in tumour production and behaviour.

This has been done in 3 ways.

1. Mice were deprived of the mammary tumour virus, or exposed to it, or exposed to one different from that carried by their own mothers, by cross-suckling on foster-mothers of a different subline or strain.

2. The genetic constitution of the mice was altered by hybridization between the sublines.

3. Mice obtained from the first 2 groups of experiments were used to study the influences of the mammary tumour virus and genetic background on the growth of transplanted pregnancy-independent tumours.

### METHODS

Both sublines have been maintained by brother-sister mating. The mice were paired at weaning and housed together throughout their lives so that *post-partum* mating could occur.

They had free access to water and diet GR25 (Dixon, Ware). Cross-suckled mice were taken from their own mother immediately after birth, or by Caesarian section, and transferred straight away to foster-mothers. The fostered young were paired at weaning and subsequently bred by brother-sister mating in the usual way.

Lines originated in this way are designated by an oblique stroke; thus T/NT indicates tumour-line mice suckled on non-tumour foster-mothers. (T $\times$ NT) $F_1$ hybrid indicates hybrids from T females and NT males.

Tumours were measured twice weekly with callipers and the product of 2 diameters recorded.

### EXPERIMENTAL

### 1. *Cross-suckling Experiments*

In these experiments T mice were prevented from obtaining the mammary tumour virus by cross-suckling on virus-free foster-mothers, either BR6 NT or C57 Black. Alternatively, they were given a different virus by using C3H foster-mothers. NT mice were exposed to a virus by cross-suckling on either T or C3H females. Table I records the number of mice with tumours in each group, out of the total number of breeding females (mice which had had at least two litters) in that group. Also shown is the behaviour of the tumours, that is whether they regressed between pregnancies or grew independently of pregnancy.

*Tumour incidence*

Evidence that a virus is essential for the development of tumours is summarized in Table I, which extends the preliminary results published by Mundy and

TABLE I.—*Tumour Incidence in Parous Cross-suckled Mice and their Progeny*

| Suckling | Foster-mother | No. with tumours/ No. $\geqslant$ 2 parous | Pregnancy | |
|---|---|---|---|---|
| | | | Dependent | Independent |
| NT | T | 52/82 | 29 | 23 |
| NT | C3H | 4/5 | 3 | 1 |
| T | NT | 0/12 | — | — |
| T | C57 | 1/24 | — | 1 |
| T | C3H | 7/19 | 2 | 5 |

Williams (1961). All BR6 (T) mice are presumed to carry the virus derived from their original male RIII progenitor, and tumour incidence in breeding females in 94%. Tumours also develop in NT mice given either BR6 or C3H virus, but not in T mice deprived of the virus.

There was 1 exception: a T/C57 female which developed a tumour when 16 months old and after 8 pregnancies. This mouse had been delivered by Caesarian section so it could have been infected with the virus *in utero*, or it may have been one of the rare occurrences of tumours arising in virus-free mice (e.g. Pullinger and Iversen, 1960), but there have been no other instances of this in our colony. As the mouse had no surviving female progeny this question cannot be answered.

The cross-suckling experiments also suggest that there may be a difference in the virus action in the fostered NT mice compared with T mice and that full expression of this action may be modified on transfer to a mouse of a different genotype. In the NT/T group, tumour incidence (63%) was significantly lower than in the T colony (94%) during the same period. There was also a low tumour incidence in the T/C3H group (37%).

*Age and parity at appearance of first tumour*

Another difference between NT/T and T females is that tumours appeared later in the cross-suckled group (Table II). However, the difference in age is not significant and is probably a reflection of the lower frequency of pregnancy

TABLE II.—*Incidence and Characteristics of Tumours in Normal, Cross-suckled, and Hybrid BR6 Mice*

| Subline | No. with tumours/No. $\geqslant$ 2 parous | Appearance of first tumour: Mean age $\pm$ s.e. | Mean parity $\pm$ s.e. | Proportion (%) of dependent tumours: All tumours | Tumours arising before 12 months of age |
|---|---|---|---|---|---|
| T | 102/109 | $7 \cdot 5 \pm 0 \cdot 4$ | $5 \cdot 4 \pm 0 \cdot 2$ | 70 | 79 |
| NT | 0/105 | — | — | — | — |
| NT/T (T × NT) | 52/82* | $8 \cdot 6 \pm 0 \cdot 5$ | $4 \cdot 2 \pm 0 \cdot 3$* | 56 | 71 |
| $F_1$ hybrid | 10/10 | $9 \cdot 1 \pm 1 \cdot 4$ | $4 \cdot 9 \pm 1 \cdot 6$ | 50 | 56 |

* Significantly different from T subline, $p < 0 \cdot 001$.

in the cross-suckled mice, which is a characteristic of the NT subline. So far the relative contributions of age and parity to tumour appearance have not been worked out.

*Pregnancy-dependence of tumours*

Both sublines of BR6 mice produced pregnancy-dependent as well as independent tumours when fostered on C3H females (Table I).

Table II shows that the overall proportion of pregnancy-dependent tumours in NT/T mice is lower than in T mice, though the differences are not significant. When only those tumours arising in mice aged less than 12 months are considered, the proportion of dependent tumours in the NT/T group is similar to that in the T group. The overall difference is therefore due to the later appearance of tumours in the NT/T mice. Fig. 1 shows that in T mice the proportion of

FIG. 1.—Influence of age on the initial behaviour of tumours in BR6 (T) mice. The number of mice in each group is given at the base of the column.

independent tumours increases with the age of the mouse at the time of tumour appearance, and tumours arising after 12 months are invariably independent, even in the few cases where they appear during pregnancy.

### 2. *Hybridization Experiments*

Two groups of $F_1$ hybrids were obtained. The first group consisted of the offspring of T mothers and NT fathers, so these mice received the virus in the usual way via the milk. The second group consisted of mice from NT mothers and virus-carrying fathers. These mice therefore did not receive virus-infected milk. The number of mice which developed tumours was recorded for each group.

*Tumour incidence*

The decrease in virus potency noticed in NT/T mice (i.e. homozygous for NT genes) was not found in a small group of heterozygous mice, as all 10 breeding females obtained from T × NT matings developed tumours (Table II).

*Transmission of virus*

The appearance of a tumour in 1 of the 6 breeding females obtained by mating NT females with virus-carrying males, suggests transmission of virus by the male. The tumour appeared very late (after 13 pregnancies and when the mouse was 15 months old), but no tumour has been recorded among 105 normal NT females (Table II) 50 of which were more than 12 months old. The tumour-bearer unfortunately had no surviving progeny so, as in the case of the cross-suckled T/C57 mouse discussed previously, the presence of the virus could not be proved.

### 3. *Transplantation Experiments*

Tumours which were growing independently of pregnancy were subcutaneously transplanted into virgin females. Donors were T females, NT/T, or (T × NT) $F_1$ hybrids. Host mice were T or NT both with and without the mammary tumour virus, so that both genetic and viral factors could be varied independently.

TABLE III.—*Growth of Transplanted Tumours*

| Donor | No. of tumours | Host | No. of transplants | No. which grew | P |
|---|---|---|---|---|---|
| T | 18 | T | 42 | 42 | < 0·001 |
| | | NT | 43 | 19 | |
| T | 10 | NT/T | 20 | 5 | < 0·001 |
| | | T/NT | 19 | 19 | |
| NT/T | 7 | T | 18 | 10 | 0·002 |
| | | NT | 19 | 19 | |
| (T × NT) $F_1$ hybrid | 4 | T | 12 | 9 | 0·1 |
| | | NT | 12 | 5 | |

The experiments summarized in Table III support the view that there is some genetic difference between the T and NT sublines. Independent tumours from T mice grew rapidly when subcutaneously transplanted into virgin T females, but only a proportion of them grew in NT females. The difference was significant ($P<0\cdot001$) and was unaffected if the T hosts were deprived of the virus (T/NT) or if the NT hosts were given it (NT/T). Similarly, tumours arising in NT/T mice were fully transplantable into NT hosts but only partially transplantable into T hosts ($P<0\cdot01$).

Tumours arising in (T $\times$ NT) $F_1$ hybrids were not completely transplantable into either parent line.

The possibility that the T subline provided a better environment than NT for the growth of incompatible tumours, was not substantiated by these results.

### DISCUSSION

These studies have established that the NT subline of BR6 mice is tumour-free because it does not carry the mammary tumour virus. As sisters of the original NT mice developed tumours, this suggests that the virus, although certainly present, was not absorbed from the milk, or was destroyed, or became modified in some way. Miroff and Magdoff-Fairchild (1965) found that destruction of the mammary tumour virus in the gut was minimal. Other instances of loss of a mammary tumour virus have been reported, for example by Andervont (1959), Murray (1963), and Smith (1966). Whatever the means of inactivation or loss of the BR6 virus, the capacity has not been inherited, as when NT mice are given the virus by foster-nursing, tumours develop.

Genetic differences between T and NT sublines have been indicated by the significantly reduced tumour incidence in NT/T mice compared with the normal T subline and by the reduced transplantability of independent tumours between the 2 sublines. Although the virus is necessary for the appearance of spontaneous tumours, growth of transplants is influenced by genetic factors rather than the presence or absence of the virus. But as Foulds (1949*b*) pointed out, the progression of a tumour could be hastened by transplantation, so that the behaviour of a transplant is not necessarily identical with that of the original tumour. Mundy and Williams (1961) also found difficulty in transplanting T tumours into NT mice although skin grafts took equally well within and between the 2 sublines. However, histocompatibility tests the homogeneity of only a fraction of the genotype and other aspects may be involved in tumour growth. Preliminary experiments suggest that transplantation failure may be due to a difference in endocrine status.

The effects of a virus may be diminished in an unfavourable genetic environment, as indicated by the significantly lower tumour incidence in the NT/T and T/C3H groups compared with the normal T subline. Andervont and Dunn (1965) noticed that after several generations the tumour incidence had fallen in some families of C3H agent-free mice foster-nursed by RIII and also in 1 family of RIII agent-free mice foster-nursed by C3H. The C3H virus was also lost from C57Bl and I strains within a few generations (Andervont, 1964).

The genetic environment also determines the behaviour of the tumour, as BR6 mice given the C3H virus developed pregnancy-dependent tumours as well as independent ones. However, Squartini, Rossi and Paoletti (1963) in cross-suckling experiments involving C3H, RIII and BALB/c strains, found that the specific tumour characteristics of the virus overrode the genetic influences for at least 6 generations.

Problems still unexplained include the reason for the loss or destruction of the mammary tumour virus, and ways in which genetic background may influence tumour development and behaviour in the presence of the virus.

### SUMMARY

Cross-suckling experiments between tumour-bearing (T) BR6 mice and a tumour-free (NT) subline showed that NT breeding females developed tumours when given the mammary tumour virus, but the tumour incidence was lower than in the T line. The tumours appeared later, and probably because of this, fewer were pregnancy-dependent. T mice deprived of the virus did not develop tumours.

Transplanted tumours grew better in hosts of the same subline as the donor, which indicates that after 45 generations of segregation there is a genetic difference between the T and NT sublines.

I am very grateful to Mr. L. E. Miall, A.I.S.T., Miss J. K. Warren and Miss M. Mosvall for skilled technical assistance and for keeping the tumour records.

### REFERENCES

ANDERVONT, H. B.—(1959) *Acta Un. int. Cancr.*, **15**, 124.—(1964) *J. natn. Cancer Inst.*, **32**, 1189.
ANDERVONT, H. B. AND DUNN, T. B.—(1965) *J. natn. Cancer Inst.*, **35**, 39.
FOULDS, L.—(1947) *Br. J. Cancer*, **1**, 362.—(1949*a*) *Br. J. Cancer*, **3**, 230.—(1949*b*) *Br. J. Cancer*, **3**, 240.
MIROFF, G. AND MAGDOFF-FAIRCHILD, B. S.—(1965) *J. natn. Cancer Inst.*, **34**, 777.
MUNDY, J. AND WILLIAMS, P. C.—(1961) *Br. J. Cancer*, **15**, 561.
MURRAY, W. S.—(1963) *J. natn. Cancer Inst.*, **30**, 605.
PULLINGER, B. D. AND IVERSEN, S.—(1960) *Br. J. Cancer*, **14**, 267.
SMITH, G. H.—(1966) *J. natn. Cancer Inst.*, **36**, 685.
SQUARTINI, F., ROSSI, G. AND PAOLETTI, I.—(1963) *Nature, Lond.*, **197**, 505.